# Recurrent and novel SS18-SSX fusion transcripts in synovial sarcoma: description of three new cases

**DOI:** 10.1007/s13277-012-0486-0

**Published:** 2012-09-14

**Authors:** Joanna Przybyl, Raf Sciot, Piotr Rutkowski, Janusz A. Siedlecki, Vanessa Vanspauwen, Ignace Samson, Maria Debiec-Rychter

**Affiliations:** 1 Department of Molecular Biology, The Maria Sklodowska-Curie Memorial Cancer Centre and Institute of Oncology, 5 W.K. Roentgen Street, 02-781 Warsaw, Poland; 2 Department of Pathology, K.U. Leuven and University Hospitals, Leuven, Belgium; 3 Department of Soft Tissue/Bone Sarcoma and Melanoma, The Maria Sklodowska-Curie Memorial Cancer Centre and Institute of Oncology, Warsaw, Poland; 4 Department of Human Genetics, K.U. Leuven and University Hospitals, Leuven, Belgium; 5 Department of Orthopedic Surgery, University Hospitals, Leuven, Belgium; 6 Postgraduate School of Molecular Medicine, Warsaw Medical University, Warsaw, Poland

**Keywords:** Synovial sarcoma, Molecular markers, Fusion genes, *SS18-SSX* fusion genes

## Abstract

Synovial sarcoma (SS) is an aggressive type of tumor, comprising approximately 10 % of soft tissue sarcomas. Over 90 % of SS cases are characterized by the t(X;18)(p11.2;q11.2) translocation, which results mainly in the formation of oncogenic *SS18-SSX1* or *SS18-SSX2* fusions. In a typical *SS18-SSX* fusion transcript, exon 10 of *SS18* is fused to exon 6 of *SSX1/2.* However, several variant fusion transcripts have been already described. In the present study, we examined the fusion transcript type in a series of 40 primary untreated SS tumor specimens using reverse transcription polymerase chain reaction and fluorescence in situ hybridization assay. We detected *SS18-SSX1* transcript in 22 (55 %) patients and *SS18-SSX2* transcript in 17 (42.5 %) patients, while in one patient, none of *SS18*-*SSX1/2* fusion transcripts were identified. Among the cases under study, two tumors carried novel *SS18-SSX1* and *SS18-SSX2* variant translocations that were allegedly created by an alternative splicing, and in additional case, an unusual translocation variant previously described by other group was found. Our data suggest that alternative splicing may play an important role in novel fusion transcript formation, and additionally we show that it may be a recurrent event in SS. Furthermore, we describe the first case of a complex rearrangement possibly linking SS to *REPS2* gene.

## Introduction

Synovial sarcoma (SS) accounts for approximately 10 % of soft tissue sarcomas. SS is an aggressive type of tumor which may arise in patients at any age but mainly develop in adolescents and young adults. SS originates principally in the extremities but may occur at any anatomic site. Metastases, which mainly affect the lungs, occur in approximately half of patients [1].

Cytogenetically, SS is characterized by the nonrandom presence of t(X;18)(p11.2;q11.2) [2, 3]. This translocation is detected in more than 90 % of SS cases and involves the *SS18* (previously known as *SYT*) gene on chromosome 18 and one of the *SSX* genes on the X chromosome [4–6]. In a typical *SS18-SSX* fusion transcript, exon 10 of *SS18* is fused to exon 6 of *SSX1/2* [7]. Approximately two-thirds of tumors carry *SS18-SSX1* translocation, and the *SS18-SSX2* variant is found in one-third of cases [5, 8, 9]. Moreover, rare cases of SS18-SSX4 chimeric variants in SS have been described. However, *SS18-SSX4* fusions have been characterized by high breakpoint variability, resulting in functional unpredictability [10–12]. The rare SS cases which lack the classical *SS18-SSX* fusion gene may represent tumors with unusual variant transcripts, which failed to be detected using conventional approaches [13]. Studies investigating the prognostic value of the different fusion types provide contradictory results. Several studies reported a more favorable outcome in patients carrying *SS18-SSX2* fusions [5, 11, 14–17], and others failed to find any significant correlation between fusion type and clinical outcome [9, 18, 19].

In the present study, we examined fusion transcript type using reverse transcription polymerase chain reaction (RT-PCR) in a series of 40 SS patients. We report two novel *SS18-SSX1* and *SS18-SSX2* variant translocations, and in one patient we detected unusual *SS18-SSX1* translocation variant previously described by other group [13].

## Materials and methods

### Patients

Forty fresh frozen surgical biopsies of primary tumors were obtained from the University Hospital Leuven, Belgium (*n* = 39) and the Maria Sklodowska-Curie Memorial Cancer Centre and Institute of Oncology, Warsaw, Poland (*n* = 1). The specimens originated from 24 males and 16 females with the mean age at the diagnosis of 38 years (range from 5 to 85 years). All tumors in our cohort were diagnosed as conventional synovial sarcomas by means of morphology and routine immunohistochemistry, with 26 tumors being classified as monophasic and 13 as biphasic. One specimen presented features of both mono- and biphasic histosubtypes. All specimens were collected with informed consent, according to the protocol approved by the local ethical committee. The clinicopathologic data of this series are shown in Table 1.

**Table 1:** Clinical, histological, cytogenetic, and molecular features of SS primary tumor specimens

No.	SS18 partner	Sex	Age	Primary site	Mitotic index/10 HPF	Histosubtype	Necrosis (%)	PFS (days)/latest status	Karyotype
1	SSX1	F	74	Wrist	35	Monophasic	No	153/DOD	74-84,X,t(X;18)(p11;q11)x2[cp14]
2	SSX1	M	26	Knee	20	Monophasic	<50	591/DOD	42-44,Y,t(X;18)(p11;q11),der(9)t(9;10)(p23;q21),-10,add(11)(p15),+12,del(14)(q10),-18,-20[cp4]/83-84 < 4n>,idem[cp3]/46,XY[3]/91 < 4n>,XXYY,inc[1]
**3**	SSX1	F	85	Shoulder	1	Biphasic	<50	328/AWOD	46,X,del(X)(p11.2),der(18)ins(18;X)(q11;p11.2p22)[11]/46,XX[4]
4	SSX2	M	46	Pharynx	36	Monophasic	<50	1931/AWD	41-45,Y,t(X;18)(p11;q11),der(1;21)(q10;q10),add(14)(q32),add(17)(q25),+19,-20[cp14]
5	SSX2	F	28	Thoracic wall	17	Monophasic	NA	548/AWD	46,X,der(X)del(X)(q23)t(X;18)(p11;q11),add(3)(p25),t(4;6)(q35;q23),t(6;12)(q21;p13),der(18)t(X;18)[15]/46,XX[4]
6	SSX1	M	17	Ankle	3	Biphasic	No	1861/AWD	49-52,Y,del(X)(q21),+der(X)t(X;18)(p11;q11)x2,t(1;2)(p22;q31),del(5)(q15),der(8)t(8;17)(p22;q21),+12,+12,add(14)(p11),del(17)(q21),-18,+20,+21[cp16]/46,XY[4]
7	SSX1	F	14	Right lower leg	2	Monophasic/biphasic	No	1276/AWD	46,X,t(X;18)(p11;q11)[9]
8	SSX2	M	47	Left kidney	54	Monophasic	No	318/AWD	45,Y,t(X;18)(p11;q11),der(1)t(1;12)(p22;q13),dic(19;20)(q13;q13)[11]/46,XY[3]
9	SSX2	M	63	Right upper leg	98	Monophasic	<50	323/AWD	73-78 < 3n+>,XYY,+der(X)t(X;18)(p11;q11)x2,+2,-3,-4,+7,+8,+9,-10,+12,+12,+12,+13,-16,der(18)t(X;18),+21[cp5]/46,XY[14]
10	SSX1	M	25	Right foot	4	Biphasic	No	672/AWOD	46,Y,t(X;18)(p11;q11)[18]
11	SSX1	M	44	Left foot	1	Biphasic	No	2573/AWOD	47,Y,t(X;18)(p11;q11),t(2;9)(q23;q34),t(3;8)(q11;q11),+8[12]/46,XY[8]
12	SSX1	M	37	Knee	23	Monophasic	NA	160/DOR	38-41,Y,-X,-3,-11,-14,-18,der(19)add(19)(p13)add(19)(q13),+dmin[cp7]/46,XY[12]
13	SSX1	M	19	Shoulder	11	Monophasic	No	256/DOD	46,Y,t(X;18)(p11;q11)[6]/46,XY[14]
14	SSX1	F	39	Upper leg	24	Biphasic	No	661/DOR	44-45,X,t(X;18)(p11;q11),t(3;12)(q27;q13),-6,der(14;21)(q10;q10),add(22)(q13),+r[cp12]
15	SSX1	F	47	Left upper leg	NA	Monophasic	NA	123/DOD	57-60 < 2n+>,XX,+X,+1,+2,+7,+9,+12,+12,+12,+14,+15,+16,+17,+21[cp15]
**16**	SSX2	M	56	Stomach	>15	Monophasic	No	399/AWD	NA
17	SSX2	M	55	Left upper leg	14	Biphasic	<50	172/DOD	35-38,Y,der(X)t(X;18)(p11;q11),-1,-2,add(2)(q37),-3,-4,add(6)(p25),-10,-11,add(12)(q24),-13,-14,-14,-15,add(16)(q24),-18,-22,add(22)(q13),+1-6mar,inc[19]/46,XY[1]
18	SSX1	M	23	Tibia	5	Monophasic	<50	2566/AWOD	44,Y,t(X;18)(p11;q11),-3,-14,-22,+r[10]/46,XY[10]
19	SSX1	F	58	Stomach	NA	Monophasic	NA	257/AWD	70 < 3n+>,XX,t(X;18)(p11.2;q11),-3,-6,+13,-14,+15,+16[15]
20	SSX1	M	49	Right groin	21	Monophasic	<50	511/DOD	54,Y,t(X;18)(p11;q11),+8,+8,der(10;13)(q10;q10),+12,+12,+15,+19,+21,+21[20]
21	Negative	F	14	Left upper leg	27	Biphasic	<50	4245/AWD	46,X,t(X;18)(p11;q11)[20]
22	SSX2	F	25	Right groin	33	Monophasic	<50	123/AWD	46,X,t(X;15;18)(p11;p11;q11)[15]
23	SSX2	F	28	Shoulder	7	Monophasic	No	2255/AWD	45,-X,der(X)t(X;?9)(q26;q13),add(16)(q22)[cp5]/46,XX[8]
24	SSX2	M	40	Lung	2	Monophasic	No	449/AWD	46,Y,t(X;18)(p11.2;q11)[2]/47,idem,+8[11]/46,XY[2]
25	SSX1	M	41	Right upper leg	22	Biphasic	<50	403/DOD	45,Y,t(X;18)(p11;q11),der(13;14)(q10;q10)c?[cp20]
26	SSX1	F	41	Left inguinal region	3	Monophasic	No	3729/AWD	46,X,der(X)t(X;18)(p11;q11)t(X;12)(q27;q15),t(1;9)(q41;q33),der(11)t(X;11)(p11;q23-24),del(12)(q15),del(18)(q11)[20]
27	SSX1	M	62	Left ankle	NA	Monophasic	NA	1981/AWD	46,Y,t(X;18)(p11;q11)[cp19]/46,XY[1]
28	SSX2	M	19	Paravertebral	28	Biphasic	NA	594/DOD	46,Y,t(X;18)(p11;q11),del(11)(q13q21)[10]/46,XY[10]
**29**	SSX1	M	33	Right knee	2	Biphasic	<50	1275/AWOD	46-52,Y,t(X;18)(p11;q11),add(3)(q26),+5,+8,+9,+14,+17,+19,i(21)(q10),+mar[cp15]/46,XY[6]
30	SSX2	F	20	Left rib	17	Monophasic	No	3572/AWD	45-46,X,t(X;18)(p11;q11)[cp19]
31	SSX1	F	26	Right upper leg	6	Biphasic	No	611/AWOD	46,X,t(X;18)(p11.2;q11),del(1)(q25),add(8)(p23),add(9)(q34)[15]
32	SSX2	M	52	Right upper leg	14	Biphasic	No	267/AWD	46,Y,der(X)t(X;18)(p11;q11),der(5)t(5;20)(p11;p11),del(12)(q22),-18,der(20)t(X;20)(p11;p11),+mar[20]
33	SSX2	F	27	Right upper leg	8	Monophasic	No	2031/AWD	46,X,t(X;18)(p11;q11)[17]/55,idem,+der(X),t(X;18)(p11;q11),+2,+4,+8,+9,+12,+19,+20,+21[3]
34	SSX1	F	15	Left upper leg	18	Monophasic	<50	351/AWOD	46,X,t(X;18)(p11;q11)[9]
35	SSX2	M	16	Right ankle	2	Monophasic	No	1717/AWOD	46,Y,t(X;18)(p11;q11)[cp10]/46,XY[10]
36	SSX2	M	57	Right upper leg	1	Monophasic	No	272/AWD	46,XY[19]
37	SSX2	F	33	Mediastinum/retrotracheal	3	Biphasic	<50	101/AWOD	NA
38	SSX1	M	77	Left upper leg	25	Monophasic	NA	243/AWD	46,Y,der(X)t(X;?3)(p11;p21),der(2)t(2;?X)(q11;p11),der(3)t(2;3)(q11;q21),inv(9)(p22q34)c?,t(10;21)(q22;q21)[20]
39	SSX2	M	49	Left lower leg	28	Monophasic	No	551/DOD	NA
40	SSX1	M	5	pharynx	50	Monophasic	<50	630/AWOD	45-47,Y,t(X;18)(p11;q11),t(7;12)(q32;q24)[cp20]

Abbreviations: *DOD* died of disease; *AWOD* alive without evidence of disease; *AWD* alive with disease; *DOR* died of other reason. Cases described in the present study are marked in bold

### RNA extraction and reverse transcription

Total RNA was extracted from fresh frozen tumor specimens characterized as SS using miRNeasy kit according to the manufacturer’s protocol (Qiagen). RNA quality was assessed using BIO-RAD Experion RNA StdSens Analysis system (BIO-RAD). One microgram of total RNA was reverse transcribed with oligo(dT)_12–18_ primers and random hexamers using SuperScript III Reverse Transcriptase (Invitrogen).

### Fusion transcript detection by PCR

We performed *SS18-SSX1* and *SS18-SSX2* detection in all of examined cases, and additionally *SS18-SSX4* detection in the case negative for *SS18-SSX1/2* fusions. PCR was performed with two microliters of cDNA using AmpliTaq Gold DNA Polymerase (Invitrogen). Amplification was performed at +94 °C for 30 s, +58 °C for 60 s, and +72 °C for 60 s for 35 cycles, and the final extension was performed for 10 min. Only the annealing temperature for *SS18-SSX4* detection was +57 °C. Primers used for amplification are listed in Table 2 [20, 21]. cDNA from synovial sarcoma 1273/99 cell line (kindly provided by Professor Fredrik Mertens, Skone University Hospital, Lund University, Sweden) was used as a positive control of *SS18-SSX2* amplification, and cDNA from patient with known *SS18-SSX1*, confirmed by direct sequencing, was used as a positive control of *SS18-SSX1* amplification. Negative controls were also included in every step of the procedure. PCR products were analyzed by electrophoresis in 2 % agarose gels stained with ethidium bromide and photographed.

**Table 2:** Primers used for PCR and sequencing

Designation	Sequence	Direction	Position	NCBI reference sequence
SS18	5′ AGGATATAGACCAACACAGCC 3′	Forward	1242-1262	NM_001007559.1
SSX1	5′ GGTGCAGTTGTTTCCCATCG 3′	Reverse	493-512	NM_005635.2
SSX2	5′ GGCACAGCTCTTTCCCATCA 3′	Reverse	510-529	NM_175698.1
SSX4	5′ GGCACAGCTGTTTCCCATCA 3′	Reverse	460-479	NM_005636.3

### Sequence analysis

PCR products were purified using QIAquick PCR Purification Kit (Qiagen). Direct sequencing of both strands of PCR products was performed using BigDye® Terminator v3.1 Cycle Sequencing Kit (Applied Biosystems) and analyzed on the 3130xL Genetic Analyzer (Applied Biosystems). Both strands of the PCR products were sequenced at least twice. Chromas Lite 2.01 software (Technelysium Pty Ltd) and BLAST software (http://blast.ncbi.nlm.nih.gov/Blast.cgi) were used for the computer analysis of obtained sequence data.

### Fusion type detection by fluorescence in situ hybridization

Fluorescence in situ hybridization (FISH) assay was performed in selected cases to confirm the type of *SS18-SSX1/2* translocations, according to the protocol described by Surace and coworkers [22].

## Results

**Fig. 1:**
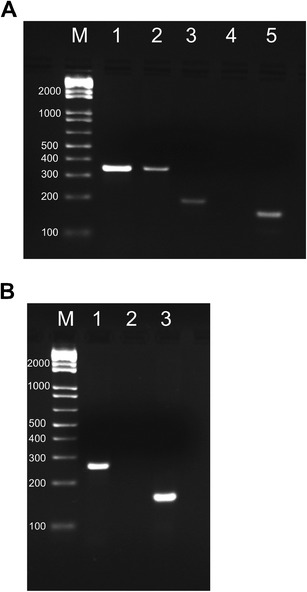
Detection of novel fusion transcripts by electrophoresis in 2 % agarose gel stained with ethidium bromide. **a** Detection of unusual *SS18-SSX1* fusion transcripts. *M*—1-kb Plus DNA ladder (Invitrogen); *1*—344-bp PCR product obtained from primary tumor specimen derived from patient #3; *2*—344-bp PCR product obtained from metastatic tumor specimen derived from patient #3; *3*—194-bp PCR product obtained from primary tumor specimen derived from patient #29; *4*—negative control, no cDNA added; *5*—positive control, PCR product obtained from patient with the *SS18-SSX1* fusion confirmed by sequencing. **b** Detection of novel *SS18-SSX2* fusion transcript. *M*—1-kb Plus DNA ladder (Invitrogen); *1*—263-bp PCR product obtained from primary tumor specimen derived from patient #16; *2*—negative control, no cDNA added; *3*—positive control, PCR product obtained from 1273/99 cell line with confirmed *SS18-SSX2* fusion transcript

We detected *SS18-SSX1* transcripts in 55 % (*n* = 22) and *SS18-SSX2* transcripts in 42.5 % (*n* = 17) of patients. In one tumor, we did not detect any fusion transcript using RT-PCR; although FISH was not performed due to insufficient material, the karyotype of this specimen was available in the hospital database, revealing a t(X;18). Among analyzed cases, we noticed larger than expected 158-bp PCR products in three patients (Fig. 1). These samples were subjected to sequence analysis. Obtained sequence data were aligned to reference fusion transcript sequences (*SS18-SSX1*—GenBank S79325, *SS18-SSX2*—GenBank S79332) and analyzed using the BLAST software. Two patients carried novel *SS18-SSX* variant translocations, and in one patient we detected atypical variant that was previously described by Amary et al. in 2007 [13].

### *SS18-SSX1* variant described by Amary et al. (2007)

An unexpected PCR product of 194 bp was detected in SS of patient #29. Direct sequencing indicated the identical *SS18-SSX1* variant transcript that was previously described by Amary and coworkers [13]. This variant includes additional 12 codons derived from exon 5 of *SSX1*, which results in the fusion of codon 410 of *SS18* to codon 99 of S*SX1* without reading frame shift. Amary et al. described this transcript variant in a 24-year-old male with a 30-mm tumor located in the right triceps. The tumor was classified as monophasic with 50 % of necrosis and 19 mitotic figures per 10 high-power fields. Patient #29 in our study was a 33-year-old male presenting biphasic a 30-mm tumor in the right knee, with less than 50 % of necrosis and two mitotic figures per 10 high-power fields. The patient underwent tumor resection directly after first diagnosis, and he was alive without disease 42 months after surgery.

### Novel *SS18-SSX1* variant

Tumor specimen #3 was obtained from an 85-year-old female with a tumor of 105-mm maximal diameter located in the shoulder. Additionally, a 30-mm metastatic tumor specimen from deep tissues of the shoulder was available from the same patient (metastasis developed 11 months after initial diagnosis). FISH analysis indicated *SS18-SSX1* fusion transcript in both of these specimens.

**Fig. 2:**
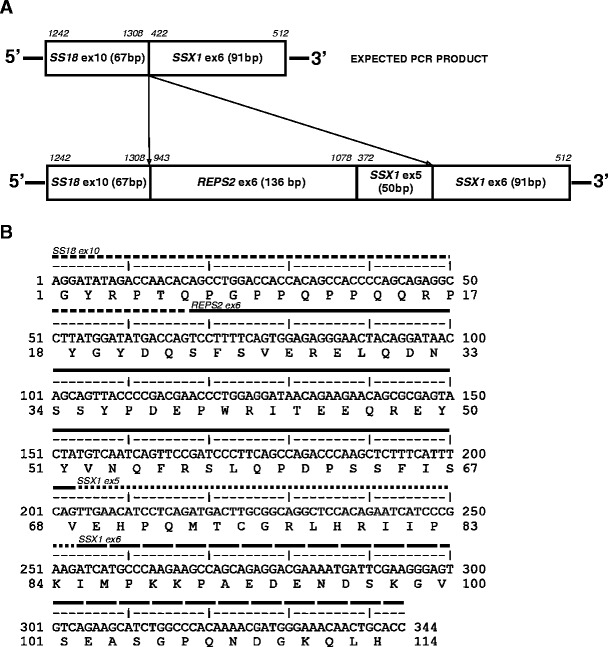
Identification of novel fusion transcript *SS18-SSX1* involving *REPS2* gene. **a** Schematic representation of the expected PCR product and novel *SS18-SSX1* fusion transcript. The nucleotide positions derived from the original sequences of *SS18*, *SSX1*, and *REPS2* mRNA are indicated in the *upper part* of the figure. **b** Nucleotide sequence of the 344-bp PCR product with predicted amino acid sequence of the chimeric protein

PCR with primers specific for the *SS18-SSX1* fusion transcript presented larger than expected products in both of the specimens. The nucleotide sequences of amplified products were identical. BLAST analysis showed an insertion of 186 bp in the site of usual *SS18-SSX1* junction. The additional nucleotides corresponded to 136 bp of complete exon 6 of RALBP1 associated Eps domain containing 2 (*REPS2*) (NM_004726.2) and 50 bp of complete exon 5 of *SSX1*, indicating a complex rearrangement in this tumor (Fig. 2). *REPS2-SSX1* insert was fused to the codon 410 of *SS18* at the 5′ end and to the codon 111 of *SSX1* at the 3′ end. This rearrangement maintained the original reading frame. The predicted chimeric protein contains 410 N-terminal codons of *SS18*, followed by 62 codons of *REPS2-SSX1* fragment (codon 46 for valine situated at the junction site of these two genes) and 78 C-terminal codons of *SSX1*.

### Novel *SS18-SSX2* variant

**Fig. 3:**
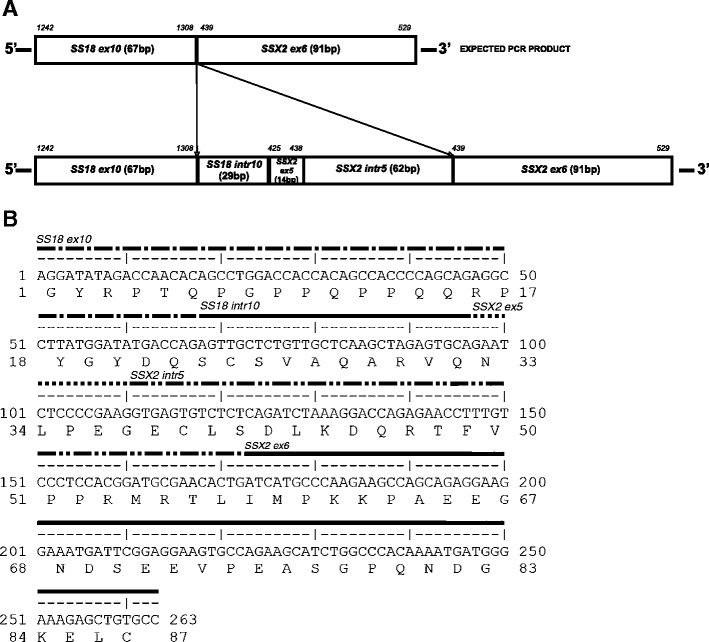
Identification of novel fusion transcript *SS18-SSX2*. **a** Schematic representation of the expected PCR product and novel *SS18-SSX2* fusion transcript. In the *upper part* of the picture, the nucleotide positions derived from the original sequences of *SS18* and *SSX2* mRNA are indicated. **b** Nucleotide sequence of the 263-bp PCR product with predicted amino acid sequence of the chimeric protein

We detected the third unusual fusion variant in a specimen #16, derived from a 56-year-old male with a 80-mm monophasic SS primary tumor located in the stomach. Patient underwent tumor resection directly after diagnosis. He developed metastasis after 13 months and was alive with disease after 42 months. FISH analysis indicated *SS18-SSX2* fusion transcript in this specimen. PCR with primers specific for *SS18-SSX2* amplified larger than expected product of 263 bp. The insertion of additional 105 bp was fused in the position of usual *SS18-SSX2* junction point. Codon 410 of *SS18* at the 5′ portion of fusion was followed by 29 bp of intron 10 of *SS18*, 14 bp of exon 5 of *SSX2*, 62 bp of intron 5 of *SSX2*, and 91 bp of exon 6 of *SSX2*, which is usually present in the 3′ end of the fusion transcript (Fig. 3). This insertion conserved the original reading frame.

## Discussion

**Fig. 4:**
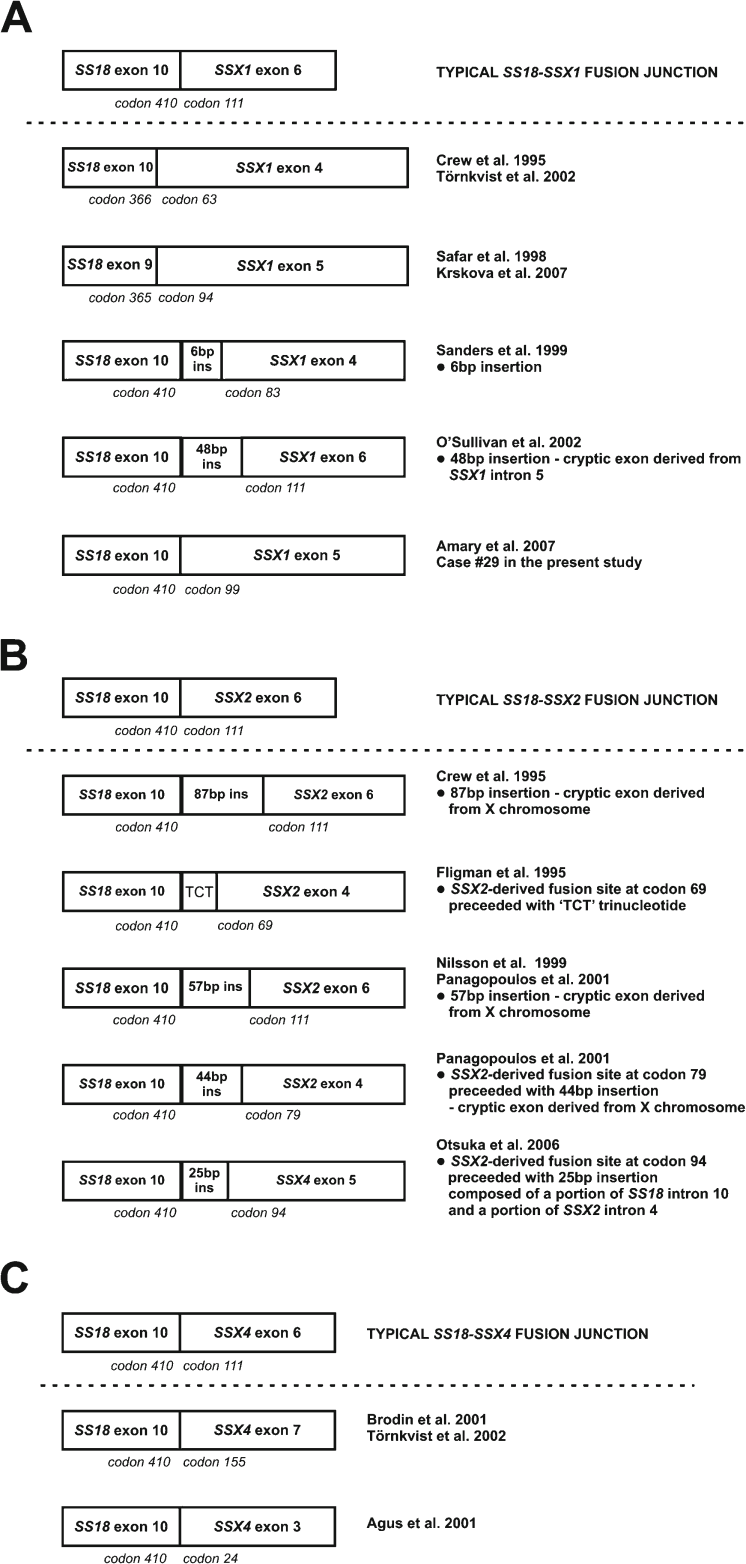
Schematic representation of novel fusion sites in the unusual transcript variants of *SS18-SSX1* (**a**), *SS18-SSX2* (**b**), and *SS18-SSX4* (**c**) described in the literature

The most common *SS18-SSX* fusion transcripts involve 410 N-terminal codons of *SS18* and 78 C-terminal codons of either *SSX1* or *SSX2*. To the best of our knowledge, there are 17 reports describing atypical variants of SS-associated translocations, which are mostly caused by small insertions [4, 7, 11–14, 23–32]. Novel *SS18-SSX* transcript variants are frequently created not only by the unusual junction of *SS18* and one of *SSX* genes but also by the mutations within *SS18* or *SSX* genes which occur apart from the fusion site, e.g., deletion or truncation of *SSX1* [26, 32] or insertion in *SS18* sequence [12]. Figure 4 presents previously reported fusion variants characterized by different *SS18-SSX* junction sites. Our examination of fusion transcript type in a series of 40 SS primary tumor specimens revealed three unusual transcript variants, two of which were not published before.

The first unusual *SS18-SSX1* transcript variant detected in our study was earlier described by Amary et al. [13]. Our detection of the identical fusion transcript variant confirms that the rare variants can be recurrent in SS patients and they are probably not caused by unique nor random events. Similar observations were already reported in several publications. Figure 4 indicates whether the atypical fusion variant was described in more than a single case.

The novel *SS18-SSX1* transcript variant was detected both in primary and metastatic specimens derived from the same patient. This translocation involved another gene, i.e., *REPS2*, also known as *POB1*, located on the X chromosome at Xp22. The product of this gene is involved in the growth factor signaling and control of cell proliferation [33–36]. The expression of this gene might serve as a biomarker of favorable outcome in breast and prostate cancers; conversely, *REPS2* downregulation has been demonstrated in the progression of prostate cancer [34, 37–39]. The fusion site of *REPS2* and *SSX1* involves amino acids encoded across a splice junction—codon 303 for glycine on the border of exons 6 and 7 of *REPS2* and codon 94 for valine at the junction of exons 4 and 5 of *SSX1*. Predicted chimeric protein sequence preserves valine in this position. Noteworthy, the junction between *REPS2* and *SSX1* was formed at the overlapping nucleotides “GGT” which are present at the 3′ end portion of *REPS2* and 5′ end portion of *SSX1*. Possibly, these overlapping nucleotides created a preferential site for the fusion of these genes. *REPS2* exon 6, which is involved in this unusual fusion, encodes a portion of the Eps15 homology domain (residues 282–373). This domain has been described to interact with Epsin, Eps15, and p65 subunit of NFκB [37, 39]. However, gene expression profiles of synovial sarcoma cases described in the present study did not show any significant differences or correlations concerning *REPS2* and its interactors (unpublished data). Therefore, the possible impact of *REPS2* gene involvement in SS-associated translocation remains unclear. Apparently, this variant preserved the transforming function of the usual *SS18-SSX1* fusion oncogene, which indicates that the mechanism of tumorigenesis in this case might have been similar as in other synovial sarcoma cases.

In addition, we have also detected novel *SS18-SSX2* fusion transcript variant that consists of exonic and intronic regions of both *SS18* and *SSX2* genes. This transcript was also presumably created by the alternative splicing mechanism since there are two cryptic “AG” acceptor sites and one cryptic “GT” donor site in the direct proximity of the fusion points. First, there is an AG signal immediately before the junction point of *SS18* exon 10 and intron 10 in position 12615–12616 of original intron 10. Second, the cryptic acceptor splice site is present immediately before the fusion point of *SS18* intron 10 and *SSX2* exon 5, involving codons 104 and 105 of *SSX2* exon 5. Finally, directly after the *SSX2* intron 5 portion involved in the fusion, there is a cryptic donor splice signal GT, which was presumably used with the authentic acceptor splice site in the 3′ end of this intron, resulting in the junction of *SSX2* intron 5 and *SSX2* exon 6.

Cryptic splicing mechanisms have previously been reported in the unusual fusion transcript formation [28]. Our data confirm that alternative splicing may play an important role in novel fusion transcript formation and that it may be a recurrent event in SS.

In our series, 64 % of the patients carrying *SS18-SSX1* translocation and 71 % of the patients with *SS18-SSX2* fusion transcript developed local recurrence or metastasis. This finding supports previous reports [9, 18, 19] showing no significant correlation between fusion type and clinical outcome.
